# On the Symptomatological Value of Palpitation

**Published:** 1851-03

**Authors:** O. B. Bellingham


*On the Symptomatological Value of Palpitation.* By Dr. O. B. Bellingham.—Dr. Bellingham thus contrasts palpitation arising from organic disease of the heart, and that independent of it.

| *Palpitation depending upon Organic Disease of the Heart.* | *Palpitation independent of Organic Disease of the Heart.* |
|---|---|
| 1. More common in the male than the female. | 1. More common in the female than the male. |
| 2. Palpitation usually comes on slowly and gradually. | 2. Palpitation usually sets in suddenly. |
| 3. Palpitation constant, though more marked at one period than at another. | 3. Palpitation not constant, having perfect intermissions. |
| 4. Impulse usually stronger than natural; sometimes remarkably increased, heaving, and prolonged; at others irregular and unequal. | 4. Impulse neither heaving nor prolonged; often abrupt, knocking, and circumscribed, and accompanied by a fluttering sensation in the præcordial region or epigastrium. |
| 5. Percussion elicits a dull sound over an increased surface, and the degree of dullness is greater than natural. | 5. The extent of surface in the region of the heart, which yields naturally a dull sound on percussion, is not increased. |

| | |
|---|---|
| 6. Palpitation often accompanied by the auscultatory signs of the diseased valves. | 6. Auscultatory signs of diseased valves are absent; bruit de soufflet often present in the large arteries, and a continuous murmur in the veins. |
| 7. Action of the heart, regular, irregular, or intermittent; not necessarily quickened. | 7. Rhythm of heart usually regular; sometimes intermittent; its action generally more rapid than natural. |
| 8. Palpitation often not much complained of by the patient; occasionally attendded by severe pain, extending to the left shoulder and arm. | 8. Palpitation often much complained of by the patient; more readily induced by mental emotion, and frequently accompanied by pain in the left side. |
| 9. Lips and cheeks often livid; countenance congested; anasarca of lower extremeties common. | 9. Lips and cheeks never livid; countenance often chlorotic; anasarca absent, except in extreme cases. |
| 10. Palpitation increased by exercise, by stimulants and tonics, etc.; relieved by rest, and frequently also by local or general bleeding, and an antiphlogistic regimen. | 10. Palpitation increased by sedentary occupations; by local and general bleeding, etc.; relieved by moderate exercise, and by stimulants or tonics, particularly the preparations of iron. |

[The influence of the coronary circulation in producing palpitation is much insisted on by the author. He says:]

In considering the immediate cause of palpitation, writers do not appear to me to have taken sufficiently into account the coronary circulation, although there can be no doubt that it performs an important part, and that derangement of its circulation will be followed by derangement of the functions of the heart equally as when the circulation through the chambers of the organ is impeded or deranged.

The integrity of a muscle in its healthy action, depends mainly, we know, upon its receiving a due supply of arterial blood; if this is sufficient, and of a good quality, the functions of the muscle will be performed with vigor; while, if the supply is too small, or if its quality is deteriorated, the functions of the muscle will be impaired. Now, the heart being a muscular viscus, requires, for the vigorous performance of its functions, an efficient supply of arterial blood: hence, although the blood which circulates through the chambers of the heart is the ordinary stimulus to the contractions of the organ, if the coronary arteries which convey the materials for its nutrition carry it in sufficient quantity, or of a deteriorated quality; or if, on the other hand, it is conveyed in increased quantity, or is of too stimulating a quality, the functions of the organ will suffer.

Thus, in anæmia, the quality of the blood being deteriorated, and a given amount containing less of the materials of nutrition, it is necessary that a greater quantity should pass through the coronary vessels in a given period, and the action of the organ is accelerated in order to compensate for the deficiency. Again, in states of plethora, where blood is rapidly formed, the heart is excited to increased action, very probably in consequence of the stimulant nature of the blood which circulates in its vessels, and its increased amount.

Lastly, in cases of valvular or other disease of the heart, where considerable obstruction exists in the pulmonary circulation, and the venous blood does not undergo the necessary changes in the lungs, the coronary arteries will convey a mixture of venous and arterial blood to the tissue of the heart; the functions of the muscle become impaired, its irritability diminishes, and it is no longer obedient to the stimulus of the blood which distends its cavities: hence its action becomes irregular and unequal; indeed, in the advanced stage of valvular or other disease of the heart where the pulmonary circulation is greatly impeded, the cessation of the heart's action, and the death of the individual, is probably owing immediately, in many cases, to the coronary arteries conveying venous blood to the tissue of the heart.

Before dismissing the subject of palpitation, I may observe, that this symptom frequently is present in a marked degree in the class of patients who present themselves at hospital, without their appearing to be conscious of it. When questioned, they allow that they feel some oppression in the præcordial region, but hardly admit that it amounts to palpitation, though, when we come to examine the chest, the action of the heart is often much increased, and its impulse sometimes so strong as to raise the head of the observer. This, no doubt, arises in some measure from the sensibility being blunted by the ill oxygenation of the blood, and in some measure, also, from the parts having had time to accommodate themselves to their altered condition. On the other hand, it is no less remarkable how often, when palpitation depends simply upon functional derangement, the patient's attention is directed mainly to it; he is most unhappy in consequence, and can scarcely be made to believe that he is not the subject of organic, and, therefore, in his opinion, of incurable disease of the heart.—*Medical Gazette.*

